# *Candida albicans* can foster gut dysbiosis and systemic inflammation during HIV infection

**DOI:** 10.1080/19490976.2023.2167171

**Published:** 2023-02-01

**Authors:** Silvere D Zaongo, Jing Ouyang, Stéphane Isnard, Xin Zhou, Vijay Harypursat, Hongjuan Cui, Jean-Pierre Routy, Yaokai Chen

**Affiliations:** a Department of Infectious Diseases, Chongqing Public Health Medical Center, Chongqing, China; b Clinical Research Center, Chongqing Public Health Medical Center, Chongqing, China; c Infectious Diseases and Immunity in Global Health Program, Research Institute, McGill University Health Centre, Montréal, QC, Canada; d Chronic Viral Illness Service, McGill University Health Centre, Montréal, QC, Canada; e Canadian HIV Trials Network, Canadian Institutes for Health Research, Vancouver, British Columbia, Canada; f Cancer Center, Medical Research Institute, Southwest University, Chongqing, China; g Division of Hematology, McGill University Health Centre, Montréal, QC, Canada

**Keywords:** *Candida albicans*, HIV, gut microbiota, dysbiosis, systemic infections, microbial translocation

## Abstract

*Candida albicans (C. albicans)* is a ubiquitous fungal commensal component of the human microbiota, and under certain circumstances, such as during an immunocompromised state, it may initiate different types of infection. Moreover, *C. albicans* continuously and reciprocally interacts with the host immune system as well as with other elements of the gut microbiota, thus contributing significantly to both gut homeostasis and host immunity. People living with HIV (PLWH), including those receiving antiretroviral therapy, are characterized by a depletion of CD4 + T-cells and dysbiosis in their gut. *C. albicans* colonization is frequent in PLWH, causing both a high prevalence and high morbidity. Gut barrier damage and elevated levels of microbial translocation are also fairly common in this population. Herein, we take a closer look at the reciprocity among *C. albicans*, gut microbiota, HIV, and the host immune system, thus throwing some light on this complex interplay.

## Introduction

There are approximately 200 known species within the *Candida* genus, and only 20 of these species have been reported to be responsible for disease development in humans ^1^. These *Candida* species are the most common human fungal pathogens, and cause (i) superficial infections on both skin and mucosal surfaces, which may be unpleasant but are relatively innocuous; however superficial infections can potentially impair an individual’s quality of life, and (ii) systemic infections resulting from the dissemination of the fungus through the bloodstream.^2^ In this context, it is known that systemic infections are life-threatening, as their mortality rates are greater than 40%.^3^ Clinical *Candida* infection rates increase yearly ^4,5^ due to the increasing number of newly-diagnosed immunocompromised patients,^6^ the widespread utilization of solid organ transplantation to treat organ failure,^7^ and the extensive and routine use of immunosuppressant drugs and broad-spectrum antibiotics, particularly in cancer chemotherapy.^8^ It is worth noting that within the 20 *Candida* species responsible for disease development in humans, the vast majority of infections are caused by *Candida albicans* (*C. albicans*),^1,9^ which is a diploid, polymorphic fungus, described as a commensal member of the normal human microbiome.^10^ Reports suggest that *C. albicans* is detected in 70%~90% of cases of candidiasis-related fungal disease.^11–13^

Among people living with HIV (PLWH), *C. albicans* is one of the most common opportunistic pathogens found during the course of HIV disease progression. It has been estimated that more than 90% of PLWH have ever developed oropharyngeal candidiasis (OPC).^14^ In one study, monitoring data derived from HIV-infected outpatients indicated that more than 50% of PLWH had been colonized by *Candida* species, and 12% of these patients developed symptomatic candidiasis.^15^ Out of the 262 strains that were isolated in the preceding study, 218 (83.2%) strains were identified as *Candida albicans* and only 44 (16.8%) strains were observed to be non-*albicans* species.^15^

In recent times, a number of studies have reported that the gut microbiota plays a major role in overall host health, and that microbial dysbiosis is purported to be associated with various diseases, including HIV, diabetes, and some cancers.^16^ As a constituent part of the normal human gut microbiota, *C. albicans* and its associated fungal virulence factors actively interact with the host immune system through adherence, invasion, and cellular damage which induces secretion of proinflammatory cytokines, and activation of antifungal activities.^17^ At the same time, there are ongoing complex interactions between *C. albicans* and other components of the gut microbiota, which impacts on the proliferation of each of the microbial components of the gut microbiota. In PLWH, *Candida spp*. are significantly more prevalent in the gut, compared to healthy controls.^18^ Moreover, PLWH also have to contend with CD4 + T-cell depletion, microbiota dysbiosis, and gut barrier damage, resulting in a high level of microbial translocation and systemic inflammation, which has previously been assumed to be one of the contributors to morbidity and mortality in this population.^19^ In this scenario, the pathogenicity of *C. albicans* is amplified, further disrupting the usually prevailing microbiological milieu of the intestine, thus enhancing gut damage and promoting microbial translocation. Herein, we review advances in the understanding of the biological and pathophysiological characteristics of *C. albicans*, its interactions with host immunity and the gut microbiota, and we critically discuss the implications of *C. albicans* infection in PLWH.

## *Candida albicans*: knowledge review

### Life cycle and epidemiology

**Figure 1.**
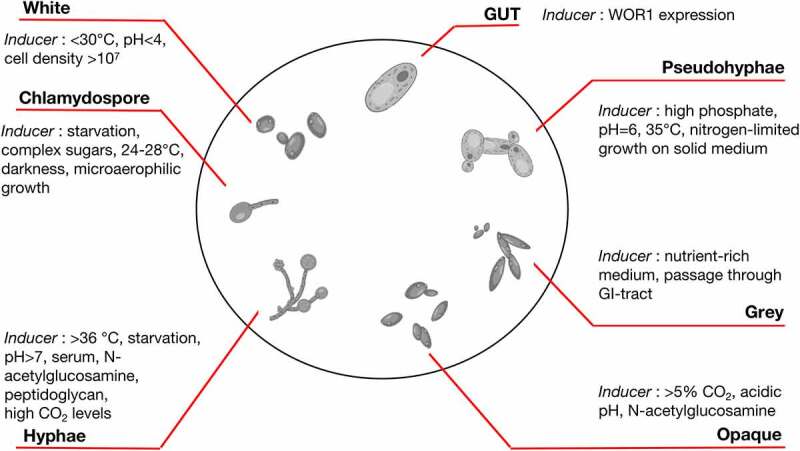
*Candida albicans* existing forms. Adapted from the review by d’Enfert et al.^26^

The genus *Candida* is classified in the family of *Saccharomycetales incertae sedis*. Evolutionary studies have indicated that one species of *Candida dubliniensis* has the closest phylogenetic relationship to *C. albicans*, with both these organisms diverging from their common ancestor (a species of *Candida tropicalis*) approximately 20 million years ago.^20,21^ Clinical isolates of *C. albicans* are generally diploid, and the genome consists of eight chromosomes, which harbors roughly 6,100 genes.^22–24^ The reference genome of the standard *C. albicans* strain, SC5314, is 14.3 megabases in length.^25^ Although no accurate and specific life cycle has been described for *C. albicans*, it is currently universally acknowledged that *C. albicans* is a largely asexual, diploid pleomorphic fungus (Figure 1), that is able to grow either as a budding yeast, as a pseudomycelium (or pseudohyphae; elongated and conjoined yeast cells), or as hyphae (formed from conjugated parallel-sided tip-growing filaments).^26^ The transition from a budding yeast to the filamentous hyphal form has been suggested to be critical for pathogenicity.^27^ Indeed, the smaller yeast cells are thought to play a role in dissemination during an infection, while the hyphal forms [triggered by expression of genes associated with virulence factors, such as hyphal wall protein 1 (Hwp1), agglutinin-like sequence protein 3 (Als3), secreted aspartic proteases 4, 5, and 6 (Sap4, Sap5, and Sap6), and hyphal-associated proteins Ece1 and Hyr1 ^28^] are more invasive and contribute to host tissue damage.^29–31^ Furthermore, *C. albicans* can manifest as a non-sexual form via a process referred to as phenotypic switching, whereby it can generate stable cell and colony variants displaying distinct properties.^32^ Such changes are spontaneous, reversible, and possibly controlled by regulatory gene expression. Based on phenotype, *C. albicans* exists in two forms: (1) white, round cells in smooth colonies and (2) opaque, rod-shaped cells in flat, gray colonies. These two forms have different tissue affinities, and antigen expression is also altered in each form. This inherent flexibility makes it highly adaptable to environmental changes.^33^ For instance, the white-GUT (gastrointestinally indUced transition) switch (adapted to survival in the digestive tract) is triggered after passage in the gut, leading to upregulation of the gene encoding for white-opaque switching (WOR1), in addition to other opaque-specific genes, as observed by Fox et al.^34^ (*in vitro*) and Pande et al. ^35^ (*in vivo*).

It is also acknowledged that *C. albicans* undergoes a parasexual life cycle, meaning that diploid cells of opposite mating types (different from conventional male/female organism forms) undergo cell fusion to create a tetraploid cell. This cellular unit subsequently undergoes a reductional division to return to a diploid state. During this division, chromosomes are randomly lost and are not exchanged in a conventional manner, although some gene conjugation occurs, to ensure genetic diversity.^36^ Furthermore, Bennett and Johnson have shown that the mating is efficient only if *C. albicans* switches from the white form to the opaque form (which is 10^6^ times more efficient for mating than the white form).^37^

*C. albicans* can successfully adapt to different organ system niches (vagina, oral mucosa, skin, respiratory tract, and/or gut) without causing disease, which suggests that the organism is highly adapted for commensalism. Indeed, it is known that *C. albicans* is carried commensally on the mucosal membranes of 67% of humans without causing any tissue damage or disease.^38^ At the same time, contemporary studies suggest that *C. albicans* is the most common serious fungal pathogen in humans, as it causes between 250,000 and 400,000 deaths per annum worldwide. Also, an estimated 100 million cases of recurrent *Candida* vulvovaginitis manifests in women throughout the world annually.^39,40^ *C. albicans* behaves like a classical opportunistic pathogen,^39^ and its presence within the host is normally under close surveillance by the host immune system and the protective bacterial microbiota in the gut or other mucosal surfaces.^41^ Due to its adaptative capabilities, *C. albicans* has the capacity to evade immune system surveillance by switching from one form to another. For example, Uwamahoro et al. have demonstrated that *C. albicans* transformation from yeast to hypha can help fungi evade phagocytosis by macrophages,^42^ and subsequently invade host tissues. Thus, the hyphal form is invariably linked to pathological processes (invasion through intercellular junctions, small ridges, and grooves associated with weakened surface/mucosal integrity, in order to take advantage of disrupted epithelial integrity ^43,44^), while the budding yeast can disseminate within the vagina or the intestine without causing overt symptoms ^45^.

### C. albicans pathogenic sites

*C. albicans* is commonly commensally found within the oral cavity, the gastrointestinal tract (GI tract), the urogenital mucosa, and on the skin. The organism may also be found in the paranasal sinus mucosa, in blood, and within various internal organs. Within each host location (Table 1), *C. albicans* must adapt to the various prevailing local environmental conditions, which differ significantly between different anatomical locations in terms of nutrient availability, pH, O_2_, and CO_2_ concentrations, etc.^61,62^ Furthermore, *C. albicans* must adapt to co-exist within and as part of the community of the individually unique microbiomes present at each anatomical location.^63^ Occasionally, however, *C. albicans* can take advantage of some of the particular conditions described in Table 1., and can become pathogenic at a specific site.

**Table 1.** Common candidiasis and their associated risk factors.

Infection Site	Study approach	Year	Population	N	Risk factors	Ref
Oral candidiasis	Case control study	2020	HIV/AIDS patients	207	Old age, male gender, xerostomia, smoking, alcohol consumption, antibiotic use, low CD4 + T-cells count, HIV infection	^45^
Oral candidiasis	Cross-sectional study	2020	HIV/AIDS patients	378	Pregnancy, oral hygiene, antibiotic use, low CD4 + T-cells count	^46^
Oral candidiasis	Retrospective cohort study	2018	Patients receiving radiotherapy for head and neck cancer	300	Lymphocyte count, severity of oral mucositis during radiotherapy	^47^
Oral candidiasis	Prospective cohort study	2013	Patients with renal transplants	1001	Mycophenolate mofetil use, dentures, tobacco	^48^
Oropharyngeal	Prospective observational study	2012	Patients in intensive care unit	110	Proton pump inhibitor use, diabetes, lower BMI	^49^
Vulvovaginal candidiasis	Retrospective cohort study	2017	Women with clinical signs of vulvovaginal candidiasis or pregnancy	2160	Pregnancy	^50^
Vulvovaginal candidiasis	Cross-sectional Study	2018	Women of reproductive age	97	Sedentary life style, wearing tights, frequent intravaginal douching, first sexual encounter when under 20 years old, number of sexual partners, curettage, not cleaning the vulva before or after sexual encounters	^51^
Esophageal candidiasis	Retrospective cohort study	2021	Immunocompetent patients	7736	Diabetes, proton pump inhibitor use, atrophic gastritis, advanced gastric cancer, gastrectomy	^52^
Vulvovaginal candidiasis	Meta-analysis	2021	Iranian women	10,536	Oral contraceptive pills use, pregnancy, antibiotics use	^53^
Candidemia	Case-control study.	2006	Pediatric patients with congenital heart disease	28	Severity of clinical condition, long antibiotic treatment	^54^
Candidemia	Case-control study	2016	Non-neutropenic critical patients	81	Long hospital stay, abdominal surgery, the use of meropenem, hemodialysis	^55^
Candidaemia	Case-control study	2021	Patients with liver cirrhosis	90	Acute-on-chronic liver failure, gastrointestinal endoscopy, long antibiotic treatment, central venous catheter, total parenteral nutrition, long in-hospital stay	^56^
Candidaemia	Case-control study	2021	Adult	118	Neutropenia, solid organ transplant, significant liver, respiratory or cardiovascular disease, recent gastrointestinal infection, biliary or urological surgery, central venous access device, intravenous drug use, urinary catheter, carbapenem receipt	^57^
Pulmonary candidiasis	Case-control study	2005	Preterm infants	20	Male gender, preterm premature rupture of membranes, duration of ventilation	^58^
Disseminated candidiasis	Case-control study	2004	Children with candidemia	153	Persistently positive blood cultures for *Candida* with a central venous catheter, immunosuppression	^59^
Invasive candidiasis	Meta-analysis	2022	Critically ill patients	1652	Broad-spectrum antibiotics, blood transfusion, *Candida* colonization, central venous catheter, total parenteral nutrition	^60^

N: Sample size; Ref: References; BMI: Body mass index.

As can be seen from Table 1, several organ systems and/or sites may be susceptible to *C. albicans* infection, thus illustrating the adaptive and immunopathological complexity and niche specificity that applies to host–pathogen interactions that mediate the commensal-to-pathogen shift by *C. albicans*. The present review focuses mainly on the presence of pathogenic *C. albicans* in the gut, and its interactions with the host gastrointestinal microbiome.

## Pathogenic *Candida albicans* in the gut: interactions with the host microbiota

*C. albicans* can become a pathogen in circumstances where nutrient availability, required pH, and CO_2_ concentrations are not necessarily ideal. Since the host gut microbiome is rich in heterogeneous carbon sources, *C. albicans* can use these alternative carbon sources to enhance its survivability and virulence.^62^ This adaptability leads to the absence of catabolite inactivation,^64^ underlining the metabolic flexibility of *C. albicans*, and also contributes to alterations of its cellular proteome and secretome,^65^ which explains its ability to switch from yeast to hyphal forms, from white to opaque units,^66^ to form biofilm, to display adhesion characteristics,^65,67^ and to remodel its cell wall.^62,68^ Remodeling of the cell wall ^62,68^ has the capacity to (i) modify the pathogen’s sensitivity to environmental stress and to antifungal drugs,^61,68^ and (ii) alter the expression and the presentation of critical pathogen-associated molecular patterns (PAMPs). Consequently, *C. albicans* becomes a continually changing target for the host immune system.^62,64^

Evidence suggests that the gut is the main reservoir from which *C. albicans* translocates through the intestinal barrier, causing bloodstream infections.^69^ Normally, *C. albicans* inhabits the intestinal tract together with other fungal and bacterial members of the microbiome, preferentially as the yeast form; however, as shown in Table 1, *C. albicans* can initiate an infection in the gut and subsequently promote gut inflammation, especially under certain specific circumstances, including during HIV infection. Thereafter, alteration of the prevailing conventional microbial and environmental equilibrium can lead to microbial dysbiosis and *Candida* overgrowth in the gut, which thus becomes a potential source for emergence and development of systemic candidiasis.^70^ Although systemic dissemination of *Candida* (occurring via transepithelial transport of the yeast form) does not rely on morphological transition, it may be mediated by indirect mechanisms such as lumen sampling dendritic cells or microfold cell transcytosis, as suggested by Vautier et al.^71^

To fully understand the impact *C. albicans* has on an individual’s (both HIV-negative and HIV-positive) immune hemostasis, a substantially more detailed and accurate knowledge of the complex interactions that *C. albicans* has with host cells and bacteria and other elements of the gut microbiome, is ultimately warranted.

### Interactions with host cells and host immune system

In the mouth, the vagina, and the gut, *C. albicans* interacts first with local epithelial and endothelial cells. Epithelial cells usually act as a passive physical barrier preventing *C. albicans* from invading through to underlying tissue.^72^ Epithelial integrity is usually maintained via tight junctions (interepithelial cell connections) which effectively seal off the gaps between the cell surface and the mucosal lamina propria, and which also prevents intraepithelial invasion of *C. albicans*.^72^ Intestinal epithelial cells also secrete protective substances (referred to as mucins) to form a mucus layer which restricts and impedes direct contact between *C. albicans* and epithelial cellular surfaces.^73^ Gastrointestinal epithelial cells can also secrete peptides or antimicrobial agents (lysozymes and β-defensins) which, combined with gastric acid, bile, and other digestive substances, work at controlling the proliferation of *C. albicans* within the gastrointestinal tract.^72^ However, two complementary mechanisms (both triggered by hyphal-associated factors ^74^) have been described with regards to *C. albicans* invasion through host epithelial cells. The initial stages of invasion are characterized by fungal-induced endocytosis, which is a process mediated by host-produced pseudopods which surround the fungal organism, and subsequently encapsulates and incorporates the fungus into the host cell. The second mechanism is the active penetration of fungal hyphae through or in between epithelial/endothelial cells.^75^ Furthermore, to overcome the antifungal influence exerted by the previously-mentioned peptides in the gut, *C. albicans* can (i) secrete peptide effectors (Sap9, Sap10, Msb2) to cleave host antifungal peptides ^76^ and inactivate a broad range of different antimicrobial peptides (AMPs) in a dose-dependent manner,^77^ and (ii) expel host antifungal peptides from the cytoplasm using efflux transporters such as Flu1.^78^

Recent investigations have shown that candidalysin, a cytolytic peptide toxin secreted by hyphae (the invasive form of *C. albicans*), is also of significant importance. Candidalysin originates from the ECE1 gene, which encodes the Ece1p protein. The Ece1p protein is then processed by Kex2p (after arginine residues are inserted at positions 61 and 93) to generate immature candidalysin. Finally, mature candidalysin is generated via Kex1p, which removes the terminal Arg93.^79^ Candidalysin is a critical virulence factor, capable of host cell activation, neutrophil recruitment, and Th17 cell-mediated immunity. It is now acknowledged that in healthy individuals, *C. albicans* which is undergoing a commensal lifestyle produces low levels of candidalysin.^79^ Then, in circumstances allowing *C. albicans* proliferation, increased candidalysin levels cause host cell and tissue damage.^80,81^ Concurrently, high levels of candidalysin activate protective innate responses through neutrophil recruitment, and Th17 immunity, altogether contributing to fungal clearance. In a similar manner, high levels of candidalysin can trigger overreactions of the immune response in certain infections (e.g., severe vulvovaginal candidiasis) and/or when the immune response is dysregulated.^82^

Once *C. albicans* is able to cope with and overcome the physical barrier formed by epithelial and endothelial cells, innate and adaptive defenses against *C. albicans* infection are required to be brought into play. The innate immune response is responsible for recognizing *C. albicans* via host cell pattern recognition receptors (PRRs) [Toll-like receptors (TLRs), C-type lectin receptors (CLRs), and Nod-like receptors (NLRs) ^83,84^] which interact with pathogen-associated molecular patterns (PAMPs) on the microbial cells (mostly cell-wall components such as β-glucan and mannan, as well as nucleic acid).^1^ Specifically, the recognition of *Candida* PAMPs is mediated by several TLRs, among which are TLR2 for the recognition of the phospholipomannan component of the cell wall, TLR4 for the recognition of short linear *O*-linked mannans, and TLR9 for the recognition of fungal DNA.^85^ This recognition (PRRs-PAMPs) therefore activates host countermeasures, including the secretion of AMPs (β-defensins, histatin, and cathelicidin LL-37), which are small molecules comprising 10–50 amino acids that are able to either kill *C. albicans* or inhibit its growth,^86^ and ultimately clear the fungal infection. However, *C. albicans* has the ability to also avoid recognition through the host PRRs by shielding its important PAMPs, such as β-glucan, with an outer mannoprotein layer of the cell wall, and therefore expose only a limited number of cell wall recognition sites.^87^ This strategy, however, renders *C. albicans* vulnerable to phagocytosis by macrophages.^88–90^ Furthermore, the innate immune system responds via epithelial cells which release proinflammatory mediators [cytokines/chemokines, particularly interleukin- (IL-) 8] in order to induce the recruitment of polymorphonuclear leucocytes (PMNs, such as neutrophils, macrophages, and dendritic cells, which are crucial for host defense against mucosal and disseminated candidiasis ^91^) from the circulating blood and direct them to the site of infection.^92,93^ According to Weindl et al., the addition of neutrophils to an epithelial model prevents both infection and cell damage by *C. albicans*.^94^ The preceding research team has demonstrated that this protective effect seems to be mediated by an upregulation of epithelial TLR4 upon stimulation with the PMN-derived tumor necrosis factor (TNF)-α. However, the precise underlying mechanisms responsible for such protection, mediated via TLR4 against *C. albicans* (and participating in the clearance of *C. albicans*), remains to be elucidated. Also, epithelial cells can themselves secrete granulocyte-macrophage colony-stimulating factor (GM-CSF), which stimulates polymorphonuclear cells and leukocytes to produce their own cytokines, including TNF-α.^75^ Once at the site of infection, PMNs can directly eliminate *C. albicans* through phagocytosis and degranulation.^95^ However, *C. albicans* does have the ability to switch from the white to the opaque form, rendering it effectively invisible to neutrophils.^96^ This invisibility subsequent to morphological ‘switching’ can be explained by the release of a chemoattractant only discovered to be present in the white form of *C. albicans*.^97^

Apart from form switching, *C. albicans* also degrades the complement factor C3b (well-known for its role in opsonization of microbial cells), and therefore inhibits recognition and uptake by phagocytic cells.^98^ However, after being phagocytosed, *C. albicans* yeasts has the capacity to morph into hyphae and thus escape from the phagocytic cell through activation of Efg1P via the cAMP/PKA pathway, resulting in the production of carbon dioxide and an alkaline pH within macrophages, for instance. This process has the potential to eventually kill the phagocytic cell, as has been demonstrated by past investigations.^99–101^ Similarly, macrophages have been found to be of particular importance with respect to fluid absorption in the colon. Indeed, Chikina et al., ^102^ observed that the innate immune system [via distal colon macrophages (CD11c^high^)] allows a rapid quality check of absorbed fluids to avoid causing toxicity to colonocytes. These specific macrophages use “balloon-like” protrusions (which positively correlates with the level of toxins) to sample the fluids absorbed through epithelial cells. In cases where the fluids are enriched with fungal metabolites/toxins (candidalysin for instance), the macrophages (through the secretion of prostaglandin 2) instruct epithelial cells to cease absorption (prostaglandin 2 induces the diminution of aquaporin localization at the apical membrane of epithelial cells ^103^), and this prevents epithelial cell poisoning and death. In the absence of this species of macrophage (with their “balloon-like” protrusions), colonic barrier integrity may well be compromised, as epithelial cells are known to undergo apoptosis subsequent to absorption of fungal metabolites/toxins. *C. albicans* interactions with innate immune cells are complex; however, dendritic cells, by phagocytosing and presenting *Candida*-specific antigens, can be considered as a link between the innate and the adaptive response to *C. albicans*.^104,105^

With respect to the adaptive response, it has been demonstrated that T-helper cells may differentiate into a specific T-helper (Th) subset depending on the nature of the antigen presented, to assist in the clearance of the infection.^106^ Indeed, the adaptive immune response can result in the induction of either the Th1, Th2, Th17, or the Treg response,^75,85,107^ depending on various different factors, which include specific molecular structures and the morphology of the fungus.^108^ Thus, each of the preceding Th phenotypes obtained from the naïve CD4+ Th precursor cell produces a distinct set of cytokines, resulting in Th cell type-specific effector mechanisms.^109^ It has now been established that the Th1 response (induced by the inflammasome, which mediates the production of IL-18 and IL-12, thus stimulating the secretion of IFN-γ, which is the prototypal cytokine of the Th1 response ^110^) and Th2 response (induced via a chitin-dependent stimulation ^88^) are more likely present during systemic mycotic infection. While the Th1 response plays a crucial role in the defense against *C. albicans* ^111^ (due to IFN-γ, which activates neutrophils, leading to pathogen elimination via phagocytosis), the Th2 response is considered to be non-protective as it (i) induces a class switch to non-opsonizing antibody subclasses and IgE ^112^ and (ii) produces IL-4, IL-5, and IL-13, which promotes allergic responses and protection against helminths.^109^ With respect to Th1ʹs proinflammatory activity, characterized by IFN-γ release, *C. albicans* has been demonstrated to be able to interfere with or modulate cytokine release, and can thus inhibit the Th1 response by avoiding recognition by TLR4.^113^ Of particular concern is the fact that *C. albicans* can, conversely, favor TLR2 activation, which drives the immune system into a non-protective Th2 response and leads to the production of anti-inflammatory cytokines. Consequently, *C. albicans* can prevent macrophage activation and also the oxidative burst used to neutralize internalized pathogens.^114^ Aside from the Th1 and Th2 responses, Th17 cells mainly assist in controlling initial growth of the pathogen and preventing invasion of tissues.^109,115^ The Th17 response can be generated via several CLRs (dectin-1, dectin-2, and mannose receptor), the NLRP3 inflammasome, the induction of IL-23, IL-1β, IL-6, and transforming growth factor (TGF)-β.^109^ Thus, the Th17 response is directed to mucosal areas (through chemokine receptors CCR6 and CCR4) where Th17 cells release IL-17A, IL-17 F, and IL-22 (Th17-characterizing cytokines) to (i) induce the recruitment of neutrophils to the site of infection and (ii) drive the production of proinflammatory cytokines, chemokines and AMPs by epithelial cells.^116^ Yet, *C. albicans* (in the hyphal form) can evade the Th17 response by releasing tryptophan metabolic byproducts (5-hydroxytryptophan metabolites, which inhibit IL-17 production).^117^ Nevertheless, Th17 cell maturation takes time, and in the quest to ensure a permanent protection against *C. albicans* in the gut, several different immune cells [such as mucosa-associated group 3 innate lymphoid cells (ILCs)] can produce IL-17 until mature Th17 cells are recruited to the site of infection. In the absence of an immune response from Th17 cells, an excessive growth of *C. albicans* can occur on intestinal mucosa. This has been demonstrated in patients with hyper IgE syndrome (in which a mutation in the transcription factor STATA3 blocks important steps of the Th17-development pathway ^109^), chronic mucocutaneous candidiasis, or an autosomal recessive deficiency of the IL-17 cytokine receptor, IL-17RA,^88^ thus highlighting the importance of the CD4 + T-cell subset in the modulation of protection against *C. albicans*.^92^

**Figure 2.**
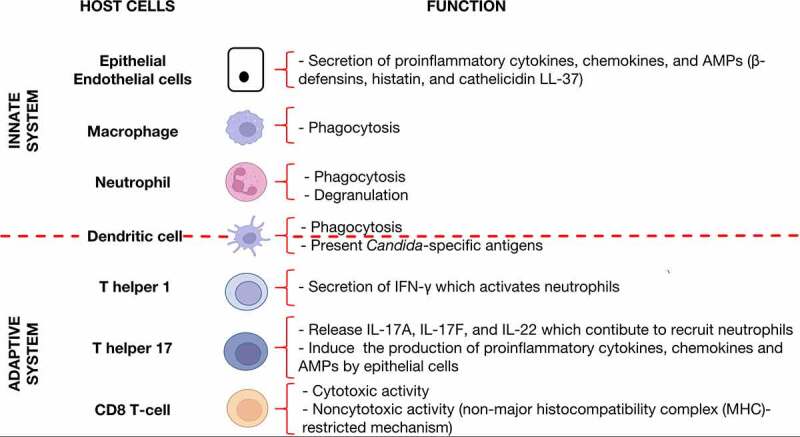
Specific host cells which contribute significantly to controlling *C. albicans* abundance in the gut.

As illustrated in Figure 2, host cells play an important role in interacting with *C. albicans*.

### Interactions with bacteria

In humans, it is generally accepted that *C. albicans* is a ubiquitous commensal component of the gut microbiome. This fungal organism resides in the intestines, within which continual and enduring inter-kingdom interactions occur with hundreds, and perhaps thousands, of bacterial taxa.^118^ Ultimately, the interactions between bacteria and *C. albicans* can be seen as interdependencies where bacterial functional and metabolic activities and products impinge upon *C. albicans*, and *vice versa*. In other words, fungi and bacteria influence the proliferation of each other in the gut. Interactions between *C. albicans* and different bacteria are summarized in Table 2, including synergistic and antagonistic effects.

**Table 2.** The interactions between *C. albicans* and bacteria.

Trend	Bacteria	Year	Model	Major findings	Ref
Synergistic	*Staphylococcus aureus*	2019	Mice	*S. aureus* can strongly adhere to *C. albicans* hyphae. Such adhesion is mediated by the Als3p protein of *C. albicans*, thereby promoting disseminated *S. aureus* disease.	^119^
		2017	*In vitro*	*C. albicans* fungal film supports the adhesion and colonization of *S. aureus* through close interaction with hyphal elements, forming a polymicrobial biofilm, and enhancing miconazole resistance.	^120^
	*Streptococcus gordonii*	2014	*In vitro*	In addition to *C. albicans’* Als3 and *S. gordonii’* SspB mediating co-aggregation between fungal and bacterial cells, Als1 was also found to bind *S. gordonii*.	^121^
		2009	*In vitro*	*S. gordonii* AgI/II proteins (SspA and SspB) mediate adhesion to *C. albicans. S. gordonii* alleviates the inhibitory effect of the quorum-sensing molecule farnesol on *C. albicans* hyphae and biofilm production.	^122^
Antagonistic	*Klebsiella pneumoniae*	2021	Mice	*C. albicans* antagonizes *K. pneumonia*, whereas *Staphylococcus spp*. may antagonize *Candida*.	^123^
	*Enterococcus faecalis*	2019	Mice	*E. faecalis* peptide EntV requires disulfide bond formation and is cleaved by proteases to produce peptides that inhibit *C. albicans* proliferation.	^124^
		2017	Mice	*E. faecalis* competes for overlapping niches by producing EntV, a 68 amino acid peptide that inhibits hyphal morphogenesis, biofilm formation, and virulence in *C. albicans*.	^125^
	*Lactobacilli*	2022	*In vitro*	*Lactobacillus johnsonii* MT4 exhibits pH-dependent and pH-independent antagonistic interactions against *C. albicans* by acidifying the local environment and producing soluble metabolites, thereby inhibiting *C. albicans* planktonic growth and biofilm formation.	^126^
		2022	*In vitro*	*Lactobacillus plantarum* inhibits the growth of *C. albicans* and *Streptococcus mutans* and disrupts *S. mutans-C. albicans* cross-kingdom biofilms.	^127^
		2013	Mice	*Lactobacilli* use endogenous tryptophan as a carbon source to amplify and produce the aryl hydrocarbon receptor (AhR) ligand, indole-3 aldehyde (3-IAld), which triggers the production of IL-22 in the gut. This results in colonization resistance to *C. albicans* and protection of the mucosa from inflammation.	^128^
	*Salmonella enterica* serovar *Typhimurium*	2011	*In vitro*	Killing of *C. albicans* filaments by *S. typhimurium* is mediated by sopB effectors.	^129^
	*Anaerobic bacteria*	2015	Mice	They limit the proliferation of *C. albican*s by stimulating the production of intestinal mucosal immune defenses, particularly cathelicidin-related antimicrobial peptide (CRAMP).	^130^
	*Streptococcus mutans*	2010	*In vitro*	Mutanobactin A, a secondary metabolite of *S. mutans*, affects the transformation of *C. albicans* from yeast to mycelium.	^131^
		2010	*In vitro*	*S. mutans* secretes trans-2-decenoic acid, a diffusible signaling factor, that inhibits filamentation of *C. albicans.*	^132^
		2009	*In vitro*	*S. mutans* production capacity-stimulating peptide (CSP) inhibits *C. albicans* embryo tube formation and yeast-to-hyphal transition.	^133^
	*Pseudomonas aeruginosa*	2013	*In vitro*	The action of phenazine produced by *P. aeruginosa* inhibits *C. albicans* filamentation, intercellular adhesion, and biofilm development.	^134^
		2004	*In vitro*	Inhibition of *C. albicans* filamentous formation by secretion of 3-oxo-C12 homoserine lactone and induction of filamentous reversion to yeast morphology in a N-acetylglucosamine-containing medium.	^135^

Ref: References

It is well recognized that antibiotic treatments induce *C. albicans* overgrowth.^136^ In an identical manner, antifungal drug administration can lead to gut bacterial dysbiosis.^137^ Shotgun metagenomic sequencing has revealed that certain bacterial strains in the human gut inhibit *C. albicans*, and this process might be controlled naturally by bacterial metabolites such as propionate or 5-dodecenoate.^136^ It also appears that *C. albicans* has the capacity to mediate significant ecological changes in gut bacterial composition,^138^ given that it has been demonstrated that *C. albicans* is actively involved in the reconstitution of gut bacterial communities subsequent to antibiotic treatment.^139^ Recently, Rao et al., ^123^ have provided a clearer understanding of the existing interdependencies between specific fungi and bacterial taxa. Indeed, they have shown that *C. albicans* antagonizes *Klebsiella pneumoniae* whereas *Staphylococcus spp*. may antagonize *Candida*. Furthermore, an article by Sun et al., ^140^ has described the existence of a positive correlation between fungal and bacterial diversity in the gut. Although they failed to reveal specific correlations between fungi and bacteria at the species level, the observations of Sun et al., suggest a mutualism between these kingdoms of organisms. Peroumal et al., using 16S and 18S ribosomal DNA (rDNA) sequence analyses,^141^ reported that colonization of the gut by *C. albicans* modulates microbiome dynamics and alleviates uncontrolled body weight gain and metabolic hormonal imbalances in a murine high-fat-diet model.

### Interactions with bacterial metabolites

Past reports have indicated that structural, functional, and metabolic products of gut bacteria may directly influence the proliferation of *C. albicans*. As such, the release of molecules such as cell surface components, peptides, or metabolic products may directly impact the biology of the fungus. Certain structural bacterial peptidoglycan (a major component of the bacterial cell wall) subunits (such as 1,6-anhydro-*N*-acetylmuramyl peptides) are highly effective hypha-inducing agents in *C. albicans*.^142^ In contrast, it has been demonstrated that EntV (a 68-amino-acid peptide), which is a product of the degradation of a 170-amino-acid prepropeptide produced by *Enterococcus faecalis* ^124^ (a commensal bacterium inhabiting the gastrointestinal tract of humans), inhibits the formation of *C. albicans* filaments, and thus prevents the formation of biofilms.^125^ Garcia et al., ^143^ have demonstrated, *in vitro*, that the human gut microbial metabolome (metabolites produced by a consortium of 60 bacterial strains derived from human feces) inhibits the proliferation of *C. albicans* to some extent, and also its ability to switch from yeast to filamentous forms. However, the identity of the specific metabolites involved as well as the identity of the actual bacterial species that produce these products are yet to be determined. Nevertheless, short-chain fatty acids (SCFAs), particularly acetic, butyric, and propionic acids have been shown to be able to inhibit germ tube formation, reduce metabolic activity in biofilms, and impair growth of *C. albicans in vitro*.^144^ Moreover, Nguyen et al., ^145^ have demonstrated that butyrate strongly inhibits yeast growth, virulence traits, and biofilm formation. Seelbinder et al., ^136^ have also demonstrated that acetic, propionic, and cis-5-dodecenoic acids reduce *C. albicans*-induced damage of cultured human epithelial cells. Also, *in vitro* evidence suggests that *C. albicans* enhances the growth of the strict anaerobes, *Bacteroides fragilis* and *Bacteroides vulgatus*.^146^ Authors of the studies reporting these findings have postulated that carbohydrates (such as α-mannan,^147^ located on the fungal surface) could fuel bacterial growth. Indeed, mannan digestion by *Bacteroides* enzymes may fuel the proliferation of *Bacteroides* species.

In addition to their direct effects on *C. albicans*, gut bacteria (via their products) can also indirectly influence the proliferation of *C. albicans*. This involves bacterial molecules (or the actual microbes themselves) which elicit responses from host cells, and the microbe-induced host response thus targets the fungus. This approach is known to be used by commensal bacteria to protect the mammalian host from invading pathogens.^148^ For example, Fan et al., ^130^ have shown that several *Bacteroidetes* and Clostridial Firmicutes restrict the proliferation of *C. albicans* by stimulating the production of gut mucosal immunological defenses, in particular the production of cathelin-related antimicrobial peptide (CRAMP).

### Interactions with other fungi

Aside from bacteria, viruses, and archaea, numerous other varieties of fungi also reside in the human gut. Fungi can produce a variety of extracellular enzymes and mycotoxins which are harmful to other microbes, including *C. albicans*. Various fungi, such as *Aspergillus, Trichoderma, Penicillium*, and yeast strains show potential antagonism against *C. albicans*.^149^ In turn, studies have also reported that *C. albicans* can inhibit various species of filamentous fungi, such as *Mucor spp* and *Chrysonilia sitophila*.^150^ Rajasekharan et al., reported that zearalenone, a mycotoxin produced by several *Fusarium* and *Gibberella* species, inhibits *C. albicans* biofilm formation and hyphal morphogenesis.^151^ Similarly, deoxynivalenol, a trichothecene mycotoxin secreted by *Fusarium*, has been observed to reduce biofilm formation and metabolic activities of *C. albicans*.^152^ Moreover, antagonistic effects of yeasts against *C. albicans* has also been reported. Indeed, Türkel et al. have demonstrated that *Metschnikowia pulcherrima* strains, isolated from grapes, have strong antagonistic activity against *C. albicans*.^153^ Murzyn et al. also reported that *Saccharomyces boulardii* yeast cells and their extract inhibit virulence factors, hyphae formation, adhesion, and biofilm development in *C. albicans*.

The preceding observations indicate that, despite the mutual effects of *C. albicans* and bacteria on each other, interactions and functional crosstalk between *C. albicans* and other commensal fungi may also play an important role in the gut microbial ecosystem. However, our understanding of the complex regulation of, and factors involved in, these interactions remains limited, especially in the gut, and particularly in the context of HIV-infected people. Much more future research is warranted to clearly elucidate the underlying mechanisms governing these intra- and inter-mycotic relationships.

## *C. albicans* immunopathogenesis during HIV infection

*C. albicans* is an integral commensal component of the healthy human gut microbiome; however, *C. albicans* can also be an opportunistic pathogen in the immunocompromised or in patients where structural and functional barriers that ordinarily prevent dissemination become disrupted or compromised.^154^ Because of its interactions with the gut microbiome, its ability to induce gut dysbiosis, and its ability to avoid immune system control, *C. albicans* infection should be considered as a potentially serious public health issue, especially in people living with HIV, where *C. albicans* pathogenicity may be significantly enhanced. In the following section, we further discuss *C. albicans* infection in the HIV-infected population.

### HIV induces immunodeficiency

**Figure 3.**
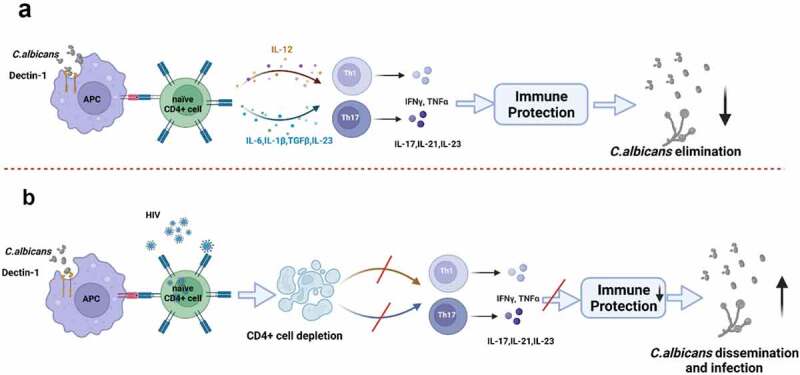
Changes of adaptive immunity for *C. albicans* in HIV-infected individuals. (a) In healthy individuals, commensal *C. albicans* colonize the host, preferably as a yeast form. They can be recognized, processed, and presented to CD4 + T cells by antigen presenting cells (APCs). Upon activation, naïve CD4+ Th precursor cells differentiate into protective effector T helper cells, Th1 and Th17, which produce multiple cytokines. These cytokines, such as IFN-γ, IL-17, and IL-21, activate immune responses to eliminate or control the growth and dissemination of *C. albicans*. (b) In HIV-infected individuals, CD4 + T-cells are invaded and depleted by HIV, especially in the intestine where CD4 + T-cells express high levels of the CCR5 co-receptor. Once CD4 + T-cell counts are reduced to threshold values, the effects of immune protection mediated by CD4 + T-cells decrease. In such a scenario, *C. albicans* would initiate overgrowth and disseminate, thus inducing different types of infection in the human host.

Once an individual is infected with HIV, their ability to competently orchestrate effective and efficient immune responses against various microorganisms becomes significantly compromised. HIV causes systemic CD4 + T-cell destruction, and results in impaired cell-mediated immunity, which leads to the inevitable emergence of various opportunistic infections,^155^ one example of which is the development of candidiasis (Figure 3). It is worth noting that *Candida* infections tend to manifest when CD4 + T-cell counts reduce to between 200 and 500 cells/µl, and may represent the first overt indication of a systemic immunodeficiency.^156^ Thus, *Candida* infections may be seen even in individuals on ART. Nevertheless, the initial step to either treating or preventing candidiasis in people living with HIV is an attempt to reconstitute the individual’s immune function by promptly initiating ART,^157^ as treating *Candida* infection in isolation in these individuals (with antifungal drugs such as fluconazole, clotrimazole, nystatin, or ketoconazole) does not either prevent recurrence or guarantee re-emergence of infection.

From an early stage of infection, HIV is known to subvert dendritic cell and macrophage activities to enhance its own replication at mucosal locations.^158^ In addition, intestinal CD4 + T-cells (which express high levels of the CCR5 co-receptor which facilitates entry of HIV virions into these cells) are significantly depleted.^159^ At both the mucosal level and within the gut, particularly, HIV contributes to progressive degradation of the immune system, and thus enables evasion by several microorganisms from routine immune system surveillance. CD4 + T-cell depletion in the gut occurs inexorably and rapidly after HIV infection,^160^ regardless of the route of exposure, and long before CD4 + T-cell reductions occur in blood or lymph nodes.^161^ Th1, Th17, and Th22 cells, which are specific subsets of CD4 + T-cells, critically important (i) for initiation of primary immune responses and (ii) for maintenance of mucosal integrity, are selectively targeted and eliminated first ^160^ (Figure 3). For example, Th1 cells (pro-inflammatory immune cells) are known to produce IFN-γ, TNF-α, and TNF-β, which primarily stimulate and promote cellular immune responses.^162^ Th17 cells, the preferential target for HIV reservoirs (as reported by Renault et al.,^163^), are abundant in mucosal-associated lymphoid tissues, and are recognized for their vital role in the immunological defense against intestinal bacterial and fungal infections.^3,164^ Th22 cells exclusively produce IL-22, and similar to Th17 cells, Th22 cells appear to have the capacity to acquire functional attributes of Th1 cells,^165^ which thus make them important immune cells for controlling *C. albicans* expansion in the gut.

In the relative absence of CD4 + T-cells (subsequent to their precipitous depletion by HIV/AIDS), CD8 + T-cells and epithelial cells become important in *Candida*-host interactions (Figure 2). Tissue biopsies from HIV positive patients [with or without oropharyngeal candidiasis (OPC)] have demonstrated an accumulation of activated CD8 + T-cells at the epithelium-lamina propria interface. However, it is worth noting that in the context of proven cases of OPC, CD8 + T-cells have been shown to be dysfunctional as they exhibit relatively small amounts of the adhesion molecule E-cadherin (which, in contrast, remain normal in OPC-negative individuals). This suggests that CD8 + T-cells may be recruited at the epithelial-lamina propria interface, where they normally have the ability to protect HIV positive individuals from developing oropharyngeal candidiasis if they normally express adhesion molecules such as E-cadherin. The preceding findings have been summarized in a past review by Fidel,^166^ who discusses the implications of HIV disease for oropharyngeal candidiasis. Instead of the recognized cytotoxic T-lymphocyte role played by CD8 + T-cells, Fidel has postulated that the effector activity of CD8 + T-cells in this context occurs via a non-major histocompatibility complex (MHC)-restricted mechanism (also referred to as the CD8 + T-cell noncytotoxic anti-HIV response),^167^ similar to a mechanism reported to be present in murine CD8 + T-cells cultured with IL-2.^168,169^ Although the preceding mechanisms have been demonstrated in the context of oropharyngeal candidiasis, they do not fully explain mechanisms which potentially occur within the gut during HIV infection. Therefore, further investigations are required in this regard.

In the absence of CD4 + T-cells the integrity of the gut cannot be ensured, and eventually leads to intestinal cell apoptosis and the progressive degradation of the tight junctions which seal the gaps between epithelial cell surfaces, both these processes contributing to the onset of leaky gut syndrome.^170^ In this context, HIV accelerates and exacerbates establishment of *C. albicans* infection, which may then easily disseminate and initiate blood stream infections. Apart from key immune cell depletion and subversion, HIV also actively participates in disruption of the natural bacterial composition and diversity within the gut, which ultimately further enhances *C. albicans* growth.

### C. albicans interactions with gut bacteria during HIV infection

In an HIV positive individual’s gut, the interactions between *C. albicans* and other microorganisms are profoundly disturbed. Indeed, a reduction in abundance of some “beneficial” bacteria, including *Akkermansia muciniphila, Bacteroides faecalis, Bacteroides vulvae, Diplococcus*, and *Arbuscular roseus* ^171^ may be noted in HIV positive individuals. Interestingly, it has been reported that potentially pathogenic microorganisms such as *Proteus, Enterococcus, Klebsiella, Shigella*, and *Streptococcus* ^171,172^ tend to become predominant in these patients. In addition, Vujkovic-Cvijin and Somsouk,^19^ in an extensive review on gut microbiome perturbations in HIV infection (covering more than twenty studies), have reported that the intestines of HIV-infected individuals are enriched with *Erysipelotrichaceae, Enterobacteriaceae, Desulfovibrionaceae*, and *Fusobacteria*, but are depleted in *Lachnospiraceae, Ruminococceae, Bacteroides*, and *Rikenellaceae*. Altogether, these changes and the atypical balance of microorganisms within the gut impact on the growth and well-being of *C. albicans*, as described above. However, it remains unclear whether the changes to gut bacterial composition subsequent to HIV infection influence *C. albicans* growth positively or negatively. Thus, further investigation will help in providing a clearer understanding of precisely what occurs in this specific scenario. Nonetheless, the knowledge gleaned from contemporary literature allows for informed speculation around this. Indeed, Rao et al., ^123^ have demonstrated that *C. albicans* antagonizes *Klebsiella pneumoniae* whereas *Staphylococcus spp*. may antagonize *Candida* in infants. We believe that these findings may not be able to be reliably extrapolated to an adult context, and furthermore, may be unreasonable to extrapolate to an HIV infection context. Thus, it is possible that during HIV infection, the existing interdependencies between specific fungal and bacterial taxa exhibit different and changing trends. Otherwise, it would be difficult to explain the relatively high proportion of *Candida* in the guts of HIV positive individuals (39.5% as reported by Awoyeni et al., ^173^ and 52.5% as observed by Esebelahie et al., ^174^) despite the predominance of potentially pathogenic bacteria such as *Klebsiella* and *Staphylococcus* within the gut. Further investigations are warranted in this specific area of inquiry.

**Figure 4.**
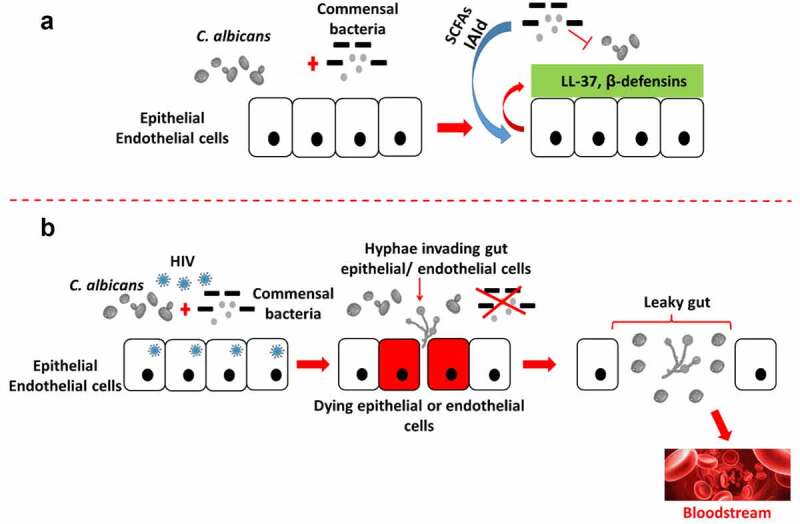
*C. albicans* interactions with host bacteria in the gut. In the absence of HIV infection, commensal bacterial actions (competition for adhesion sites, secretion of IAld and SCFAs) combined with host epithelial/endothelial cells secretion of β-defensins and LL-37 contribute to control *C. albicans* proliferation (a). However, during HIV infection, the reduced abundance of commensal bacteria favors *C. albicans* proliferation, which in turn develops hyphal forms and biofilm on and in-between epithelial/endothelial cells. Consequently, epithelial/endothelial cell integrity is disrupted, leading to their destruction, which further contributes to the establishment of the leaky gut syndrome (b). The epithelial/endothelial disruption eventually represents an “open gate”, so to speak, for *C. albicans* to disseminate into the bloodstream and thus initiate systemic infection and inflammation. SCFAs, short-chain fatty acids; IAld, indole-3-aldehyde.

Results from previous investigations have pointed out the critical role of *A. muciniphila* in the gut, supporting intestinal mucosal homeostasis by modulating mucus thickness,^175^ leading academics to consider *A. muciniphila* as a potential “sentinel of the gut”.^176^ Indeed, *A. muciniphila* has the ability to (i) stimulate mucin production within the gut, (ii) improve enterocyte monolayer integrity, (iii) counteract inflammation, and (iv) induce intestinal adaptive immune responses.^177,178^ During HIV infection, a significantly lower intestinal abundance of *A. muciniphila* has been observed in PLWH. However, this is not an established conclusion, as the results of metagenomic studies have been heterogeneous, and not all of these studies have observed a reduction in this specific bacterial species in PLWH. Nevertheless, the lower abundance of *A. muciniphila* depends neither on whether or not ART treatment has been initiated, nor on prevailing CD4 + T-cell counts or viral loads. To our knowledge, there is no study in the published literature which clearly explains the possible mutual reciprocity existing between *A. muciniphila* and *C. albicans*, especially during HIV infection. However, in the light of available information gleaned from contemporary literature, it is possible to speculate on a negative association between these organisms, meaning that when *A. muciniphila* is abundant, *C. albicans* growth is inhibited, and vice versa. Indeed, since a major role of *A. muciniphila* is to prevent bacterial translocation in the context of the leaky gut syndrome, it is difficult to conceive of a positive association between these two organisms knowing that pathogenic *C. albicans*, in order to establish and disseminate infection, is likely to induce the leaky gut syndrome, as explained by Hofs et al.^88^ However, further investigations are required to clearly establish this possibility. In the relative absence of commensal bacteria (as seen during HIV infection), *C. albicans* is able to colonize epithelial surfaces and form hyphae. The subsequent inter- and intracellular invasion, therefore, facilitates the release of hydrolytic enzymes which profoundly damage epithelial cells, resulting in the loss of epithelial integrity (Figure 4).

Despite the evidence presented in the previous paragraphs, it is important to understand that the diversity and composition of the gut microbiome is also intimately linked to sexual preference. A study by Noguera-Julian et al., ^179^ has shown that men who have sex with men (MSM) have a *Prevotella*-rich enterotype, while non-MSM men are enriched in *Bacteroides*. In the preceding study, HIV negative MSM have been reported to possess a significantly richer and more diverse fecal microbiome compared to HIV negative non-MSM subjects. The preceding study also reported that HIV infection induced a diminution of bacterial richness in both categories of people (i.e., MSM and non-MSM). This said, we believe that the predominance of *Bacteroides* (which include *Bacteroides faecalis and Bacteroides vulvae*, well-known for their commensal role in the gut) in non-MSM subjects may indicate their predisposition to superior gut integrity, compared to MSM. In this regard, *C. albicans* proliferation in the non-MSM gut may likely be under relatively more stringent surveillance, compared to gay men. Therefore, much deeper investigation will be necessary to understand the biology of *C. albicans* in the gut (HIV-positive or not) based on sexual preference. Notwithstanding sexual preference, Rosas-Plaza et al., ^180^ have independently demonstrated that industrialized populations present less bacterial diversity in the gut microbiome than that from non-industrialized populations. Notably, *Bacteroidaceae, Lanchospiraceae*, and *Rickenellaceae* are predominant in the gut of populations living in modern societies. Their study involved comparing data collected from 14 studies, which represented the gut microbiome of 568 individuals from hunter-gatherers, agricultural, agropastoral, pastoral, and urban populations. Furthermore, they were able to establish that different lifestyles foster a distinct bacterial population diversity in the gut, and this supports the hypothesis that urbanization influences bacterial diversity in the human gut microbiome. Thus, urban populations and rural populations are likely to possess distinctly different microbiomes. In this regard, the influence of *C. albicans* on the gut of HIV positive individuals from either rural and urban populations remains to be further studied and clarified.

### C. albicans takes advantage of microbiota dysbiosis and favors microbial translocation in PLWH

Normally, in an HIV negative individual, *C. albicans* may become pathogenic and colonize the gut subsequent to antibiotic drug treatment. Antibiotics non-selectively eliminate commensal bacteria that may inhibit the overgrowth of *C. albicans* through (i) a competition for adhesion sites, (ii) the secretion of inhibitory molecules [indole-3-aldehyde (IAld), SCFAs and lactic acid for example, which can inhibit the transition from yeast to hypha, and ensure a low environmental pH], and (iii) the induction of specific secretions from epithelial cells, such as β-defensins and LL-37. Thus, *C. albicans* is free to proliferate and switch from yeast to hyphal forms expressing adhesins/invasins, which are essential in the process of adhesion to and invasion of epithelial cells. In addition, *C. albicans* produces hydrolytic enzymes such as secreted aspartic proteases (Saps) to further facilitate the invasion of epithelial cells, to critically damage cells, and compromise epithelial integrity.^88^ In the absence of effective treatment, a progressive leaky gut syndrome is established. This scenario can be likened to *C. albicans* being a hidden ‘time-bomb’ waiting for a favorable opportunity to wreak havoc on the human body. Indeed, in the HIV infection context, in addition to impairment of the immune system’s ability to comprehensively respond to infections, there is a relative reduction of the abundance of commensal bacteria, including SCFA-producing bacteria (particularly the *Ruminococcaceae* and *Lachnospiraceae* taxa, which comprise the primary producers of SCFA).^19^ Furthermore, the bacterial microbiome of ART-treated PLWH can be depleted due to administration of preventive antibiotic drug treatment,^181^ especially in those with low CD4 + T-cell counts (also referred to as immunological nonresponders). Consequently, in the gut, a reduction of total SCFA production may promote the activation of intestinal T-lymphocytes, enhancing HIV replication, and the induction of further immune cell depletion.^182^

The microbial dysbiosis and the severe gut-associated immune cell depletion that takes place in the gut during HIV infection are now recognized to be associated with a degraded intestinal barrier, impaired mucosal immunological function, increased microbial translocation, and long-term immune activation in PLWH.^19,176,183^ In other words, HIV itself induces gut-associated microbiota dysbiosis, and increases susceptibility to the development of leaky gut syndrome. HIV infection therefore favors the pathogenicity of *C. albicans*, which in turn can easily exacerbate damage to the gut epithelial barrier and promote microbial translocation. Indeed, as HIV infection progresses, *C. albicans* (i) has no further competition for adhesion sites, (ii) can easily proliferate, and (iii) can proceed unchecked with its invasion of the epithelial barrier (Figure 4). HIV positive individuals are consistently found to have evidence of microbial translocation, even those on ART, with a suppressed viral replication and load.^184,185^ As such, microbial translocation markers such as LPS remain substantially high after ART initiation, compared to HIV-negative individuals.^186^ With the combined influence of *C. albicans* proliferation and HIV infection, the circulating levels of such markers cannot return to normal. Furthermore, it has been demonstrated that plasma LPS and bacterial DNA levels in ART-treated patients inversely correlate with CD4 + T-cell reconstitution in the gut-associated lymphoid tissue (GALT).^187,188^ Thus, we speculate that therapeutic interventions to block microbial translocation in ART-treated patients may be important for complete reconstitution of gastrointestinal immunity. To illustrate this, it is pertinent to refer to the immune status of immunological nonresponders, who maintain high levels of microbial translocation and immune activation, and fail to restore their CD4 + T-cell population, despite long-term ART,^187,189^ suggesting that microbial translocation can potentially thwart the likelihood of ART success. Thus, *C. albicans* in the gut can, indeed, be regarded as a mycopathological ‘time-bomb’ which may jeopardize immune reconstitution and also ART success in HIV positive individuals.

Finally, the influence of HIV on distal colonic macrophages (CD11c^high^) and their “balloon like” protrusions, as defined by Chikina et al., ^102^ as regulators of fluid absorption by the colon, is worth exploring. Infection of these cells by HIV is likely to (i) decrease the population of these specific macrophages, (ii) dysregulate their protrusion formation, (iii) dysregulate the rapid quality check of absorbed fluids in the colon, (iv) induce toxicity of colonocytes by candidalysin generated by *C. albicans*, and (v) favor epithelial cell death (apoptosis). In such a context, *C. albicans* will further degrade gut integrity, increase microbial translocation, and aggravate the HIV-positive individuals’ clinical state.

#### Conclusions

To conclude, it appears from study of the available literature that the immunopathogenesis of *C. albicans* in humans has now been relatively well described. Overt *Candida* infections, resulting from pathological activity of *C. albicans* in particular locations of the body, are inextricably linked to interactions and interdependencies of *C. albicans* with other members of the local communal microbiome. In this article, we have explored how *C. albicans*, a commensal colonizer of most humans, and an opportunistic pathogen in the immunocompromised or in patients in which barriers that prevent dissemination have been disrupted, can evade immunological and commensal bacterial surveillance and regulation, to become pathogenic. As such, *Candida* represents an ongoing threat to every individual. In the particular context of HIV infection, where both the immune system and commensal bacterial populations are profoundly destabilized, *C. albicans* can therefore be considered to be a mycological ‘time-bomb’, waiting the perfect moment to strike and exacerbate microbial dysbiosis, leading to the leaky gut syndrome, subsequent microbial translocation, and the undesirable and potentially deadly consequences thereof. Thus, *C. albicans* should be considered as an important public health threat, even in this era of affordable and widely available ART, as microbial translocation may prevent the reconstitution of gastrointestinal immunity and the overall success of ART treatment. Lastly, we believe a close monitoring of *C. albicans* abundance within the gut of HIV positive individuals, particularly at the immunological level (using CD4 + T-cell counts, CD4+/CD8+ ratios, and measures of chronic systemic inflammation), and its associations, correlations, and interdependencies with both pathogenic and non-pathogenic commensal microbiome in the gut could assist in fostering a more comprehensive understanding and prevention of the consequences of *C. albicans* translocation in humans.

## Data Availability

Data sharing is not applicable to this article as no new data were created or analyzed in this study.
